# Quinine in Uterine Conception

**Published:** 1875-06

**Authors:** J. S. Wetherly

**Affiliations:** Montgomery, Ala.


### QUININE IN UTERINE CONCEPTION.

By J. S. WETHERLY, M.D., Montgomery, Ala.

A great many articles have been written within the last five years upon the ecbolic effects of quinine. Nearly all of these articles, if my memory serves me correctly, were written for the purpose of proving that quinine does act upon the pregnant uterus in such a way as to force it to expel its contents; and that, consequently, it should not be given to females who are pregnant, or if given at all, it should be given with great caution, even if the patient be suffering from malarial fever. This opinion has been communicated to the laity by physicians, creating a prejudice against its use in certain cases.

I have been induced to write this paper from the fact that I fear great harm is likely to result from classing this indispensable drug under a head to which I am satisfied, from personal observation, that it does not belong.

False facts in medicine are worse than false theories. We have enough of both. I have been prescribing quinine for the last twenty-five years, and I am satisfied that it will much oftener arrest uterine action than it will produce it. I do not mean that it will control active labor pains, but in those irregular or neuralgic pains which are frequently produced by malarial poisoning, a full dose, or several full doses, will almost invariably arrest them, just as morphine will do under the same circumstances. The woman rests, and also the womb; and if it is about her full time, when the pains return, she will probably be delivered without pain and immediately. Reasoning from wrong premises, it is announced that quinine or morphine has acted as an ecbolic, when, in fact, the action has been the very opposite.

Some years since, this question was up for discussion before our local society, and a committee consisting of Drs. J. B. Gaston, W. O. Baldwin and R. F. Michel was appointed to experiment with quinine upon pregnant animals. Dr. Gaston has kindly furnished me with the following report of the experiments, and also of some cases treated by himself about the same time:

MONTGOMERY, ALA., Sept. 3, 1874.

*J. S. Wetherly, M. D.:*

DEAR DOCTOR: The experiments made in 1870, of which you have requested a statement, were as follows:

1. Six grains of sulphate of quinine were given to a white woman, three months advanced in pregnancy, every two hours until thirty grains were taken. The same quantities were given at the same intervals on the following day. There were no uterine contractions, no hæmorrhage, and no other perceptible effects except the indications of intense cinchonism. This woman was married, the mother of two children, and about twenty-eight years old.

2. Ninety grains of sulphate of quinine were given to a negro woman, two and a half months advanced in pregnancy, in three successive days—thirty grains each day in doses of six grains at intervals of two hours. Effects the same as in the preceding case.

The aforesaid women were very willing subjects of experiment, and I watched them closely, ready at any time to suspend the quinine if uterine pain had been excited.

3. A committee of three—Drs. J. B. Gaston, W. O. Baldwin and R. F. Michel—appointed for the purpose by the M. M. and S. Society, made a series of experiments on a pregnant bitch, as follows:

(*a*) Ten grains of sulphate of quinine, in solution, were injected hypodermically.

(*b*) Fifteen grains of sulphate of quinine were administered in the same way.

(*c*) Twenty grains were administered as before.

These, and other similar experiments with still larger quantities of quinine, were made on different but not always successive days, with no other than the locally irritant and generally poisonous effects of quinine, until they killed the animal.

Yours, etc., J. B. GASTON, M.D.

I could furnish quite a number of cases occurring in my own practice during the last twenty-five years, but will content myself with the report of four cases which I think ought to be regarded as typical:

About two years since a professional friend asked me to see his wife, stating that she had been suffering from an attack of intermittent fever for about ten days; that she was some months advanced in pregnancy; that she had miscarried several times at or about the seventh month, and that she was now very seriously threatened with premature labor. He also said that he had refrained from the use of quinine during this attack of fever from the fact that he believed it excited uterine contractions. He and his wife were both anxious that she should go to full term and be delivered of a living child. Instead of quinine, he had been using morphine freely, also chloral, and some domestic remedies for the purpose of preventing the daily occurrence of the chill and fever, none of which were, however, successful, and the exacerbation came on regularly every day, and with each exacerbation an increase of uterine pains. On seeing the patient, I found her with high fever, very restless, notwithstanding that she was completely under the influence of opium. Strong uterine pains were coming on every few minutes. On inquiry, I found that these pains came on with every exacerbation of fever, but that they were much more severe on that evening than any time previous. She informed me herself that she knew she was in labor. It was now 8 P.M., and I found uterine pains coming on very regularly every five or six minutes. On making an examination per vaginam, I found the os uteri dilated to the size of a silver half-dollar, soft, and still dilatable. I felt the child's head presenting covered by the membranes. I gave it as my opinion that the only agent that would be likely to prevent a premature delivery was quinine. Two other physicians were present with the husband, all entertaining the same opinions as to the ecbolic effect of quinine. They all objected to my prescription at first, but finally consented that I should have my way that night. I consequently ordered,

℞.—Quinine, . . . . . . . . grs. xx
Extract Hyos. . . . . . . . grs. iv,

To be divided into two powders; one to be given at 12 o'clock at night, and the other one at 6 next morning. She took the prescription as directed, and the next day she had no fever or pains.

The following night the same prescription was repeated, and three grains of quinine ordered every morning afterwards until frost. She had no more trouble, came to full term, and was delivered of as fine a specimen of a boy as I ever saw. I feel perfectly sure that this lady could not have had another paroxysm of fever without delivery having taken place.

About two months since I was called to see Mrs. S, of this city. She was six months advanced in pregnancy. I found her suffering with what I at first supposed to be an attack of catarrhal fever, attended with quite severe uterine pains. I treated her with anodynes, without benefit, for two or three days, when I became satisfied that she had remittent fever; the uterine pains getting worse with every paroxysm of fever. At my afternoon visit (I think on the third day of her sickness), I found her suffering with severe and regular uterine pains; the uterus could be felt through the abdominal walls, getting hard with every pain. A digital examination proved the os to be dilated sufficiently for the index finger to pass readily through the os, coming in contact with the head of the fœtus. I informed her husband that she would probably be delivered that night. I ordered quinine, grs. xx., and extract hyosceamy, grs. iv., to be divided into two powders, one to be given at 12 o'clock and the other at 6 A.M.

The next day was passed in comparative comfort. The same prescription was repeated the two following nights with the effect of subduing both fever and pains. I have no doubt she will go to full term. She has continued to take the powders at intervals ever since her first attack, one or two of them always being sufficient to control the uterine pains, which have continued to annoy her at intervals ever since the first attack.

Soon after seeing this case, I was called to see a colored woman seven months advanced, who had been quite sick for several days with an attack of intermittent fever; she had, however, no uterine pains. I gave her the same prescription mentioned above. Her intermittent was soon controlled, and she was ordered to take quinine daily until she was confined.

Two weeks since I was called to a woman who said that she was within two weeks of her confinement. She had quite a high fever with great tendency to congestion of the brain. She had had but one chill. I prescribed thirty grains of quinine for her, to be given at 12, 4 and 8 A.M. She had no more fever, and was delivered a day or two since at full term.

My opinion is that fevers of any kind are apt to excite uterine action; that it is especially the case with malarial fevers; and if they are not promptly relieved the uterus will expel its contents, whether quinine has been given or not. The uterine action is, however, never due to the quinine, but is the effect of the disturbed circulation and the malarial poison itself.—*Richmond and Louisville Medical Journal.*

